# The association between life events and mental health among adults in Java, Indonesia: Investigating the moderating effects by education, asset index, and rural-urban area of residence

**DOI:** 10.1371/journal.pone.0348726

**Published:** 2026-05-18

**Authors:** Sri Idaiani, Mashita Fajri, Irmansyah Irmansyah, Jonathan Gibson, Jack Wilkinson, Herni Susanti, Helen Brooks, Penny Bee, Asri Maharani

**Affiliations:** 1 Research Centre for Preclinical and Clinical Medicine, National Research and Innovation Agency, Cibinong, Indonesia; 2 Faculty of Nursing, Universitas Indonesia, Jakarta, Indonesia; 3 Research Centre for Public Health and Nutrition, National Research and Innovation Agency, Cibinong, Indonesia; 4 Division of Population Health, Health Services Research & Primary Care, University of Manchester, Manchester, Lancashire, United Kingdom; 5 Division of Nursing, Midwifery and Social Work, School of Health Sciences, The University of Manchester and Manchester Academic Health Science Centre (MAHSC), Manchester, Lancashire, United Kingdom; Universitas Syiah Kuala, INDONESIA

## Abstract

Life events (LE) are significant experiences (e.g., bereavement, illness, or job loss) that require psychological adjustment and may contribute to mental health disorders like depression and anxiety. Indonesia faces a rising burden of these conditions, yet limited research has explored their relationship with LE in its diverse socio-cultural context. This study examines the associations between LE, depression, and anxiety among adults in Java, Indonesia. It further explores how these associations vary by education, asset ownership (proxy of household wealth) index, and rural-urban area of residence. We used cross-sectional data from the baseline of the Sustainable Treatment and Care for Anxiety and Depression (STAND) 2023 longitudinal household survey. The study included 19,186 participants aged 18 years and older. Depression and anxiety were assessed using the CES-D-10 and GAD-7 scales, respectively, both validated for this population. LE was measured using the Social Readjustment Rating Scale, which categorises exposure into low, moderate, and high-stress levels over the past 12 months. Logistic regression was used to evaluate the associations, adjusting for demographic, socioeconomic, lifestyle, and health-related factors. Both primary models (main effects only) and interaction models (to explore moderation) were fitted. Overall, 4.4% of respondents reported depressive symptoms, and 8.5% reported anxiety. In the primary models, experiencing moderate or high levels of LE was significantly associated with depression (AOR = 3.1, 95% CI: 2.6–3.6; AOR = 10.2, 95% CI: 8.0–12.9, respectively) and anxiety (AOR = 2.7, 95% CI: 2.5–3.1; AOR = 6.5, 95% CI: 5.3–8.0, respectively). These associations were moderated by socioeconomic factors. Higher education, a higher asset index, and urban residence were associated with lower odds of anxiety and depression. Interventions should prioritise mental health literacy and access to care, particularly in rural and socioeconomically disadvantaged populations.

## Introduction

Depression and anxiety are among the most prevalent and rapidly increasing mental health conditions worldwide. Recent evidence from the Global Burden of Disease Study 2023 indicates substantial increases in age-standardised disability burden since 2010, particularly for anxiety disorders (62.8%) and depressive disorders (26.3%), placing them among the leading causes of years lived with disability (YLD) globally [1]. Globally, depressive disorders ranked 2nd and 11th, while anxiety disorders ranked 3rd and 12th among other diseases calculated through YLD and DALY (Disability Adjusted Life Year) in 2023 [1]. These trends are especially concerning in low- and middle-income countries (LMICs), where more than 80% of people with common mental disorders reside, accounting for approximately 8.8% of the total disease burden [1]. In these settings, rising non-communicable disease burdens, including mental health conditions, intersect with constrained health system capacity and declining development assistance for health [2].

In Indonesia, recent estimates from the Global Burden of Disease Study indicate that 8.3 million people live with depressive disorders (3.1% prevalence) and 12.4 million with anxiety disorders (4.6% prevalence) in 2021. These conditions also contribute substantially to disability, with depressive disorders accounting for 1.28 million DALYs and anxiety disorders for 1.49 million DALYs in this region [3]. In contrast, age-standardised prevalence rates for these disorders tend to be higher in high-income countries due to better detection and reporting, though the absolute burden remains disproportionately large in lower-resource settings like Indonesia [4]. This high burden is exacerbated by lower income levels, limited access to mental health services, under-resourced healthcare systems [5] and population ageing [6]. In addition, recent bibliometric work on Indonesia’s primary healthcare system has highlighted that while external opportunities for strengthening services exist, internal capabilities, particularly human resources and infrastructure, remain limited [7]. Beyond structural and systemic challenges, individual exposure to stressful life events is also recognised as a major determinant of depression and anxiety [7].

Live events (LE) is an experience that has an identifiable onset and ending, but has the potential to change a person’s mental and physical condition‌‌ [8]. Although LEs are time-bound, their psychological consequences can be long-lasting, particularly when they involve severe disruptions such as the death of a loved one [9], job loss [10], or exposure to violence [11]. Using GBD data, Liu and colleagues identified intimate partner violence, bullying victimisation, and childhood sexual abuse as key life events associated with depression [5]. Previous studies further show that such events may not only trigger the onset of depression [12] and anxiety, but also prolong recovery and worsen prognosis among individuals with existing depression or anxiety disorders [13]. Among the few available LMIC studies, research from South Africa found that stressful life events, including partner violence, relationship stress, and social strain, were significantly associated with mood and anxiety disorders, with stronger associations observed among women and those with lower educational attainment [14]. Similarly, a systematic review of studies from Brazil, Chile, India, and Zimbabwe found that common mental disorders were consistently associated with negative life events, poverty, and rapid social change [15], suggesting that the mental health consequences of LE may be particularly pronounced in resource-limited contexts where social protection and mental health services are scarce.

A key theoretical framework linking life events and mental health is the stress–diathesis model, which suggests that stressful events may trigger depressive or anxiety symptoms in individuals with pre-existing vulnerabilities, such as genetic predisposition or social disadvantage [16]. The impact of LEs, however, is not uniform; their psychological consequences can vary depending on individual and contextual factors, including socioeconomic conditions. The stress–diathesis model suggests that mental disorders result from the interaction between environmental stressors and individual vulnerability, which may be biological, psychological, or social in nature. A more detailed description of the stress–diathesis framework is provided in S1 Appendix.

Informed by the stress–diathesis framework, socioeconomic position may influence how individuals experience and cope with stress. In this study, socioeconomic context is examined through multiple indicators, including education, asset index, and rural–urban residence, which shape exposure to stressors as well as access to material, psychosocial, and environmental resources. Education impacts mental health by shaping employment prospects, income stability, and the ability to develop effective coping skills [17,18]. Household wealth affects mental health more in the low-education group, influencing it through insurance investment and labour supply, impacting individuals both in the short term and long-term [19]. Measuring household economic status poses particular challenges in low- and middle-income countries (LMICs) such as Indonesia, where reliable data on income and expenditure are often limited [20]. As a result, asset-based measures have been widely used as proxies for household wealth [21,22]. In this study, we therefore use an asset index as a proxy for wealth, given its suitability for capturing welfare in developing country settings and its greater stability compared with income or expenditure measures [23]. In addition, places of residence, such as urban and rural areas, impact individuals differently. Cities offer more facilities and conveniences, whereas rural regions tend to provide greater social support [24].

Evidence from LMIC on the relationship between LE and mental health remains limited, with much of the existing evidence derived from high-income settings, including studies from the United States [9], Europe [25,26], and Hong Kong [27], as well as a systematic review predominantly drawing on high-income country samples. Research examining the relationship between LE and mental health outcomes, particularly depression and anxiety, has identified several important patterns but also notable gaps. First, although studies on LE have been widely published, many focus on specific populations, such as individuals with long-term physical health conditions (such as cancer, diabetes, and obesity) [28–33] or discrete demographic sub-groups such as students and adolescents, or pandemic situations [34–36]. Moreover, most studies come from high-income countries, which may not account for the unique sociocultural, economic, and environmental factors affecting LMIC populations. For instance, Sanchez et al. demonstrated how ecological domains influence the relationship between life events and depressive symptoms among adolescents, indicating the importance of context [37]. However, studies focusing on LMIC populations are scarce, making it difficult to apply these findings universally or to understand how life events differ based on cultural perspective [38].Second, evidence on LE and mental health in Indonesia specifically is limited. To date, only one identified study has examined this relationship, focusing on peripartum women rather than the general adult population [39]. Related studies nonetheless suggest that life stressors are highly relevant in the Indonesian context, including elevated depression among older adults with limited income and assets [40], high psychological distress among health workers in remote settings [41], and increased mental disorder risk following disaster exposure, such as the 2006 Yogyakarta earthquake [42]. These findings underscore the need for population-based studies that explore how life events influence depression and anxiety in Indonesia.

Third, the role of socioeconomic factors as moderators of the LE–mental health relationship remains underexplored, particularly in LMIC settings. Previous studies have examined the individual effects of socioeconomic factors, such as education [43], wealth [43,44], and rurality [45] on mental health outcomes, suggesting that these factors may modify vulnerability or resilience to stress. However, few studies, particularly in LMIC settings, have explicitly tested whether socioeconomic factors moderate the relationship between life events and mental health outcomes. In Indonesia, such moderation analyses remain largely unexplored, limiting understanding of how social and contextual inequalities influence stress-related mental health risks.

To address these gaps, we conducted a large, population-based study in Java, Indonesia to investigate how exposure to life events relates to mental health in an LMIC context. Using data from the STAND Indonesia survey [46], which was designed and implemented by the study team, we examined two study aims. The first study aim examined whether LEs were independently associated with depression and anxiety among adults in Java, after accounting for key sociodemographic characteristics and broader contextual factors. The second study aim assessed whether the association between LEs and mental health outcomes modified by education, asset index, and rural–urban residence. By identifying how sociodemographic and contextual factors modify the mental health impact of life events, this study contributes to a more nuanced understanding of risk and resilience, with implications for both prevention and policy.

## Methods

### Study design and setting

**Fig 1:**
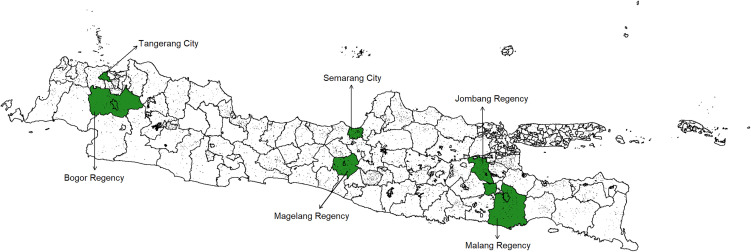
Geographic Distribution of Study Sites in Banten, West Java, Central Java, and East Java, Indonesia. Map redrawn by the authors using openly licensed geographic boundary data from openfreemap.org; the figure is distributed under the Creative Commons Attribution (CC BY 4.0) licence‌‌.

This study used the 2023 survey dataset from the Sustainable Treatment and Care for Anxiety and Depression in Indonesia, which employed a cross-sectional design and was conducted on Java Island, specifically in Banten Province, West Java Province, Central Java Province, and East Java Province. We did not include the Special Regions of Jakarta and Yogyakarta because these two provinces have special characteristics, namely Jakarta as the nation’s capital and Yogyakarta as a sultanate. Data collection was conducted from July 27 to October 20, 2023. The survey was conducted in Cipadu Village (Tangerang City, Banten Province); Gunung Bunder 1, Gunung Bunder 2, and Cibunian Villages (Bogor Regency, West Java); Bulu Lor and Bandarharjo neighbourhoods (Semarang City, Central Java) and Treko and Borobudur Villages (Magelang Regency, Central Java); and Bululawang and Karangsari Villages (Malang Regency, East Java) as well as Sumberaji and Dukuhklopo Villages (Jombang Regency, East Java). The selection of villages was based on the representativeness of urban and rural areas. The representatives of the urban area were Gunung Bunder 1, Cipadu, Borobudur, Bandarhardjo, Bulu Lor, and Dukuhklopo, while the representatives of the rural area were Gunung Bunder 2, Cibunian, Treko, Sumberaji, Kasembon, and Karangsari. The geographic distribution of the study sites is presented in Fig 1.

### Sample size

This study is part of a larger programme of research, and the overall sample size of the survey was calculated based on objectives relating to the feasibility of implementing the survey longitudinally. Based on these objectives, the target sample size was calculated as 4,800 households, representing approximately 19,200 individuals. This sample size provides adequate power for the study objectives. Based on previous regional surveys, the prevalence of depression or anxiety is estimated at approximately 10% [47]. Under this assumption, a sample size of approximately 1,250 participants per village would be sufficient to detect associations between life events and anxiety or depression corresponding to an odds ratios of 1.5 or greater (i.e., an increase in risk of anxiety or depression from 10% to 15%) [48]. After accounting for a modest design effect of 1.2 (typical for large community surveys), the required sample size increases to approximately 1,500 participants per village. The achieved sample size of 1,600 participants per village therefore exceeds this requirement.

### Data collection

#### Study participants.

The study population comprised adults aged 18 years and older living in villages/*kelurahan* across four provinces of Java. The selection of regencies/cities, sub-districts, and villages/*kelurahan* was based on an agreement between the research team and the Province and District Health Office. Within selected villages/*kelurahan*, RTs (Rukun Tetangga or neighbourhood units) were chosen by first listing all RWs (Rukun Warga or community units) and RTs. The number of RTs to be included was determined using the formula n = 1600/number of individuals aged ≥18 years × number of RTs in the village/*kelurahan*. RTs were then selected using random sampling with Stata version 17. All eligible residents, defined as permanent residents aged 18 years or older and willing to participate, were subsequently invited by enumerators for interviews.

#### Survey administration.

Data collection was preceded by the recruitment and training of enumerators (data collectors), who were selected based on relevant qualifications and experience in survey implementation. The fieldwork was carried out by the Faculty of Nursing, Universitas Indonesia, in collaboration with the University of Manchester. The preparation and design of the questionnaire, as well as the overall data collection protocol, were developed by researchers from both institutions. Research assistants supported the preparation and administrative aspects of the study and served as liaisons between the researchers, field coordinators, and enumerators. Field coordinators supervised the enumerators, who were responsible for conducting the household interviews in the selected study sites. Prior to field deployment, all enumerators underwent comprehensive training covering the objectives of the study, ethical procedures, interviewing techniques, and the use of digital tools for data entry.

The data collection process began after enumerators provided participants with an explanation of the study and obtained written informed consent. This explanation was conducted one day before the interview. Interviews were administered face-to-face, and responses were entered directly into mobile devices using a Computer-Assisted Personal Interview (CAPI) program.

The structured questionnaire, administered in Bahasa Indonesia, included questions about location information, individual and household demographics, household expenses, health conditions, outpatient and inpatient care, medical costs, quality of life, history of chronic illnesses, depression, anxiety, loss of productivity, social networking, and life events. Other survey modules, including quality of life measures, social network and mental health service utilisation, were not analysed in this manuscript and are reported elsewhere [46]. A survey was considered complete if respondents answered all questions from the demographic section through the life events module. Each interview took approximately 30 minutes.

#### Response rate.

A total of 20,160 individuals were invited to participate in the survey, of whom 19,236 consented, yielding a very high response rate (95.4%). Nonetheless, the possibility of non-response bias cannot be entirely ruled out. Most non-participation occurred because potential respondents were not at home during the survey period, despite being provided with an explanation sheet several days beforehand. To further minimise bias, we compared the characteristics of respondents whose data were excluded due to substantial missing information with those of respondents included in the analyses. The results showed no significant differences in the characteristics of the respondents analysed.

### Measurements

#### Depression.

Depression was measured using the Centre for Epidemiologic Studies Depression Scale (CES-D) questionnaire. This questionnaire consists of 10 questions with Likert scale responses ranging from 0 to 3. The questions inquire about experiences related to depressive symptoms over the past week. The total score ranges from 0 to 30. Depression was categorised based on an ordinal scale: a score of 0 indicates “never” or ≤1 day per week; 1 indicates “rarely” or 1–2 days per week; 2 indicates “sometimes” or 3–4 days per week; and 3 indicates “often” or 5–7 days per week. During analysis, positive items are reverse-scored. Higher total scores indicate a greater likelihood of depression. In this study, scores were dichotomised using a cut-off of 10: participants scoring 10 or above were classified as experiencing depression, and those scoring below 10 as not depressed [49]. Previous studies using CES-D in Indonesia were small-scale and focused on specific populations, such as adolescents or caregivers [50,51]. During the preliminary analysis of this study, factor analysis and reliability testing were conducted, showing that the factors were consistent with the original questionnaire and yielded a Cronbach’s alpha of 0.70.

#### Anxiety.

General Anxiety Disorder questionnaire 7 (GAD-7) was used to assess anxiety. The validity and reliability of this questionnaire has been conducted in prior study [52]. The GAD-7 consists of questions about symptoms of anxiety experienced over the past two weeks, with response options being “0 = not at all,” “1 = several days,” “2 = more than half the days,” and “3 = nearly every day.” Therefore, the total score ranges from 0 to 21. Based on the total score, we divided into binary groups: those with anxiety and those without. The cut off was ≥ 6 for anxiety therefore the variable is a binary [53]. In Indonesia, the GAD-7 was initially translated and validated for epilepsy patients [54]. Until now, the validation study for GAD-7 for general population has not been available. According the preliminary analysis of this study, the factors were also consistent with the original questionnaire and yielded Cronbach’s alpha of 0.92.

#### Life event.

The measurement of life events was based on the Social Readjustment Rating Scale (SRRS) created by Holmes and Rahe in 1967 [55], which include 43 life events commonly associated with psychological stress. The SRRS has demonstrated good reliability, with coefficients ranging from 0.96–0.89 in control groups and from 0.91–0.70 in psychiatric groups. Additionally, the weights (values) of all life events are fairly consistent, ranging from 0.83 to 0.59 for control groups [56].

Each life event in the SRRS is assigned a weight value ranging from 1 to 100, with the highest value of 100 representing the death of a spouse, and the lowest value of 11 representing a minor violation of the law, such as traffic tickets, a fine, or disturbing the peace [55]. The SRRS conceptualises life events as major changes that require readjustment, regardless of whether the events are typically perceived as desirable or undesirable. Respondents are asked to complete a checklist of life events experienced in the past 12 months. The total score is calculated by summing up all the life events that have been experienced. Scores of 150 or less are categorised as low, scores between 150 and 300 are categorised as moderate, implying about a 50% chance of a major stress-induced health problem in the next 2 years. Scores of 300 or higher are categorised as high, which corresponds to about an 80% chance of a major stress-induced health problem in the next 2 years, according to the Holmes-Rahe prediction model [57].

The SRRS has undergone many adaptations and revisions by various researchers, reflecting evolving needs and developments [57–60]. For this study, the original SRRS created by Holmes and Rahe is used [55]. Although the instrument was translated into Bahasa Indonesia and reviewed for clarity and conceptual equivalence by bilingual experts.

#### Moderating variables.

This study also examines whether the relationship between life events and mental health outcomes varies across different key sociodemographic groups, defined by education, asset index, and rural-urban residence.

Education: Education was categorised into three levels based on the highest level of formal schooling completed by the respondent: primary education (no schooling, incomplete or completed primary school), secondary education (incomplete or completed junior and senior high school), and tertiary education (diploma, undergraduate, or postgraduate degrees). This classification allows for the analysis of educational attainment as a social determinant of mental health, reflecting both access to cognitive resources and potential coping mechanisms in the face of life stressors.

Asset Index: Wealth was assessed using an asset index based on questions regarding property and household equipment ownership. Previously, Ariawan created an asset index using the 2013 Indonesian Basic Health Research (*Riset Kesehatan Dasar*), which included ten variables to construct the index [61]. We followed the same steps and found that during principal component analysis (PCA), not all variables successfully contributed to forming a reliable index. Only four variables were considered valid, as the analysis showed that the index explained 56% of the variation in ownership of the four types of items and housing conditions.

The four variables with eigenvalue greater than one were house walls, toilet facilities, refrigerators, and televisions. The variables for house walls and toilets are measured on an ordinal scale: bamboo or woven bamboo is rated 1, wood, boards, or triplex are rated 2, and brick or similar materials are rated 3. For defecation facilities, rivers, seas, lakes, or bushes are rated 1, private toilets without septic tanks and public toilets are rated 2, and private toilets with septic tanks are rated 3. Ownership of televisions and refrigerators is measured on a nominal scale (Yes = 1 or No = 0).

In the final stage, asset scores were summed and categorised into two groups based on the observed score distribution. Category 1 represents households and individuals with the lower asset index, while Category 2 represents those with the higher asset index. This categorisation reflects the limited variation in asset scores within the study sample and ensures a stable and interpretable measure of household socioeconomic position.

Rural-urban area of residence: The classification of urban or rural areas is based on a list compiled by the Central Bureau of Statistics (BPS-Statistics Indonesia). This classification follows the BPS Head Regulation No. 37 of 2010 on urban and rural classification [62]. This classification considers population density, the percentage of agricultural households, and the presence/accessibility of urban facilities within a village or sub-district. Urban facilities assessed include the availability of kindergartens, elementary and secondary schools, markets, shops, cinemas, hospitals, hotels, billiards/dance clubs/discotheques/massage parlours/salons, the percentage of households with telephones, and the percentage of households with electricity. Each facility is assigned a score, and the total score is calculated. An area is classified as urban if the total score is ≥ 10, and rural if the total score is < 10.

### Covariates

The covariates included age [63], gender [64], employment [65] and marital status [66]. Age play a significant role in mental disorders [67] and was categorised into 18–24 years (reference), 25–34 years, 35–44 years, 45–54 years, 55–64 years, 65–74 years, and ≥75 years. Sex is dichotomised into male and female. Employment was categorised as employed and unemployed. Marital status was categorised divided into single, married, and widowed (which includes both divorced and separated). The participants’ provinces of origin are categorised into Banten, West Java, Central Java, and East Java.

### Statistical analyses

Before conducting the main analysis, we performed data cleaning and excluded records with missing values on key variables. In the initial analysis stage, univariable and bivariable analyses were conducted to describe respondent characteristics, including chi-square tests for categorical variables.

The first study aim assessed whether LEs were independently associated with depression and anxiety among adults in Java, Indonesia. Given the binary outcomes (depression and anxiety), we applied logistic regression to estimate associations with life events while adjusting for covariates. Each model included LEs as the primary independent variable and adjusted for key sociodemographic covariates: education level, age group, sex, marital status, and province of residence. Adjusted odds ratios (AORs) and 95% confidence intervals (CIs) were reported to quantify the association. Model fit was assessed to ensure the robustness of the estimates.

The second study aim examined whether the association between life events and mental health outcomes varied by education, asset index, and rural–urban residence. For each potential moderator, separate logistic regression models were fitted that included an interaction term between LEs and the moderating variable. We conducted a series of analyses incorporating different covariate variations: (i) without interaction, (ii) interaction between LEs and education, (iii) interaction between LEs and rurality, and (iv) interaction between LEs and asset index. These interaction terms allowed us to explore how specific contextual factors might modify the relationship between LEs and mental health outcomes.

To aid interpretation, we present marginal effects from the logistic regression models, which estimate the predicted change in the probability of experiencing depression or anxiety associated with each explanatory variable, holding other variables constant. Results were visualised using the ‘margins’ command in Stata to illustrate the adjusted probabilities of mental health outcomes across levels of LEs and interaction terms.

This study contained some missing data, which may affect the precision of estimates. To minimise bias from incomplete information, we employed multiple imputation [68]. This approach uses all available predictors to generate plausible values for each missing observation, thereby accounting for uncertainty in the imputation process. To assess robustness, we conducted sensitivity analyses by comparing results obtained from the imputed datasets with those derived from the original data. The statistical software used for the analysis was Stata version 17.

### Ethic statements

All procedures followed were in accordance with the ethical standards of the Ethics Committee of the Faculty of Nursing, Universitas Indonesia, Indonesia, with number KET-237/UN2.F12.D1.2.1/PPM.00.02/2022, and the Ethics Committee of the National Research and Innovation Agency (BRIN), Indonesia, with number 010/KE.03/SK/11/2022. Informed consent was obtained from all participants for being included in the study.

To ensure informed and voluntary participation, the consent procedure allowed individuals ample time to decide. Enumerators initially visited potential participants at their homes to distribute the Participant Information Sheet (PIS), then provided a 24-hour period for them to consider their involvement. On the following day, the enumerators returned to follow up. If the individual agreed to take part, they were asked to sign an informed consent form prior to the interview, upholding ethical standards throughout the survey process.

## Results

Of the 19,236 respondents, 50 records were excluded from the analysis due to more than 70% of the variables being missing (major incomplete data), leaving 19,186 individuals for the final analysis. Table 1 illustrates the descriptive statistics of the respondents and compares their characteristics according to depression and anxiety. The mean age of the respondents was 43.33 ± 16.51 years. On average, more than half of the respondents were female (55%) and had attained at least a secondary education (49.3%). Most of the respondents were married (74.1%) and employed (55.3%), with a nearly equal distribution between urban and rural areas. Most respondents were classified in the higher asset category. Additionally, the number of significant life events experienced in the past year was generally low.

**Table 1:** Descriptive baseline characteristics and proportion of depression and anxiety.

Variables	Total (N = 19,236)*	Depression (N = 845) **	Non-Depression (N = 18,315) **	p	Anxiety (N = 1,627) **	Non-Anxiety (N = 17,544) **	p	Missing data (%)
**Age (year)**								0.13
18-24	2,691 (14.0)	7.1 (6.2-8.1)	92.9 (91.8-93.8)	<0.001	11.8 (10.6-13.1)	88.2 (86.9-89.4)	<0.001	
25-34	3,705 (19.3)	3.7 (3.1-4.3)	96.3 (95.6-96.9)		8.48 (7.6-9.4)	91.5 (90.6-92.4)		
35-44	4,064 (21.1)	3.3 (2.8-3.9)	96.7 (96.1-97.2)		8.81 (7.7-8.9)	92.0 (91.0-93.6)		
45-54	3,957 (20.6)	3.4 (2.9-4.0)	96.5 (95.9-97.1)		7.53 (6.7-8.4)	92.0 (91.6-93.2)		
55-64	2,791 (14.5)	4.5 (3.8-5.4)	95.4 (94.6-96.2)		7.2 (6.3-8.3)	92.8 (91.7-93.7)		
65-74	1,488 (7.7)	5.4 (4.4-6.7)	94.5 (93.3-95.6)		8.71 (7.4-10.3)	91.3 (89.7-92.6)		
≥75	515 (2.7)	8.3 (6.2-11.1)	91.6 (88.9-93.7)		8.36 (6.3-11.1)	91.6 (88.9-93.7)		
**Sex**				0.006			<0.001	0.04
Male	8,643 (44.9)	4.0 (3.6-4.4)	96.0 (95.6-96.4)		7.2 (6.6-7.7)	92.8 (92.3-93.3)		
Female	10,586 (55.0)	4.8 (4.4-5.2)	95.2 (94.8-95.6)		9.56 (9.02-10.14)	90.4 (89.9-91.0)		
**Education**				0.805			0.919	0.06
Primary	8,569 (44.6)	4.51 (4.1-5.0)	95.5 (95.0-96.0)		8.43 (7.86-9.04)	91.6 (91.0-92.1)		
Secondary	9,484 (49.3)	4.38 (4.0-4.8)	95.6 (95.2-96.0)		8.56 (8.02-9.15)	91.4 (90.9- 92.0)		
College	1.171 (6.1)	4.09 (3.1-5.4)	95.9 (94.6-96.9)		8.29 (6.84-10.01)	91.7 (90.0-93.2)		
**Marital status**				<0.001			<0.001	0.04
Single	2,758 (14.4)	7.08 (6.2-8.1)	92.9 (91.9-93.8)		11.2 (10.1-12.5)	88.7 (87.5-89.9)		
Married	14,204 (74.1)	3.5 (3.2-3.8)	96.5 (96.2-96.8)		7.7 (7.3-8.2)	92.2 (91.8-92.7)		
Widowed	1.806 (9.4)	7.0 (5.9-8.3)	93.0 (91.7-94.0)		9.3 (7.9-10.5)	90.9 (89.4-92.1)		
Divorced	411 (2.1)	5.8 (3.9-8.5)	94.1 (91.4- 96.0)		11.95 (9.1-15.5)	88.0 (84.5-90.8)		
**Employment**				<0.001			<0.001	0.04
Employed	10,613 (55.3)	3.7 (3.3-4.0)	96.3 (95.9- 96.7)		7.4 (6.9-7.9)	92.6 (92.1-93.1)		
Unemployed	8,568 (44.7)	5.3 (4.8 −5.8)	94.7 (94.2-95.1)		9.8 (9.2-10.5)	90.2 (89.5-90.8)		
**Residence**				0.100			0.007	0
Urban	9,625 (50.0)	4.2 (3.8-4.6)	95.8 (95.4-96.2)		7.9 (7.4-8.5)	92.0 (91.5-92.6)		
Rural	9,611 (50.0)	4.7 (4.3-5.1)	95.3 (94.9-95.7)		9.0 (8.5-9.6)	91.0 (90.4-91.5)		
**Province**				<0.001			0.152	0
West Java	4,803 (25.0)	3.1 (2.7-3.7)	96.8 (96.3-97.3)		8.7 (8.0-9.4)	91.3 (90.5-92.0)		
Banten	1,624 (8.4)	2.4 (1.7-3.3)	97.6 (96.7-98.2)		8.1 (7.4-8.9)	91.8 (91.0-92.6)		
Central Java	6,403 (33.3)	4.5 (4.0-5.1)	95.5 (94.9-95.9)		8.8 (8.2-9.6)	91.1 (90.4-91.8)		
East Java	6,406 (33.3)	5.8 (5.2-6.4)	94.2 (93.6-94.7)		7.3 (6.1-8.6)	92.7 (91.4-93.9)		
**Asset index**				0.002			0.219	0.2
Lower asset	5,959 (31.1)	5.1 (4.6-5.7)	95.0 (94.3-95.4)		8.8 (8.2-9.6)	91.1 (90.4-91.8)		
Higher asset	13,227 (69.0)	4.1 (3.8-4.4)	95.9 (95.5-96.2)		8.3 (7.9-8.8)	91.7 (91.2-92.1)		
**Life event**				<0.001			<0.001	0.2
Low	14,220 (74.1)	2.7 (2.5-3.0)	97.3 (96.9-97.5)		5.9 (5.6-6.4)	94.1 (93.7-94.4)		
Moderate	4,389 (22.9)	7.7 (6.9-8.5)	92.3 (91.5-93.1)		14.3 (13.3-15.4)	85.7 (84.7-86.7)		
High	577 (3.0)	20.1 (17.1–23.6)	79.9 (76.4-83.0)		27.1 (23.6-30.9)	72.9 (69.1-76.4)		

Notes: *presented are n (%); ** presented are % and 95% Confident Intervals, missing values on CES-D = 76 individuals (0.4%); missing values on GAD-7 = 65 individuals (0.3%).

The percentage of individuals experiencing depression was 4.4%, while those experiencing anxiety amounted to 8.5%. The prevalence of depression was highest among the oldest group ≥75 years (8.3%; CI: 6.2–11.1%) and younger individuals aged 18–2 (7.1%; CI: 6.2–8.1%), while anxiety was more prevalent among the group 35–44 years (8.8%; CI: 7.7–8.9%) and the group 65–74 years (8.7%; CI: 7.4–10.3%). Both depression and anxiety were more common in women compared to men. Individuals with higher levels of education tended to have lower rates of depression and anxiety compared to those with lower education attainment. Depression was more frequently experienced by individuals who were single and widowed, while anxiety was more frequent in single and divorced individuals. Similarly, a higher proportion of unemployed individuals experienced these conditions compared to those who were employed. In this study, residents of rural areas were more likely to experience both depression and anxiety. By province, the highest proportion of depression was found in East Java, whereas the highest rate of anxiety was observed in Central Java. Consistent with existing evidence, depression and anxiety were more prevalent among individuals with fewer possessions. Depression was more prevalent among those who had experienced moderate life events, while anxiety was more prevalent among those who had experienced significant life events.

Tables 2 and 3 show the relationship between LE and depression and anxiety. Models 1a, 2a, and 3a are the primary models reporting main effects, while Models 1b, 2b, and 3b are interaction models exploring moderation by education, asset index, and rural-urban residence, respectively. The following results are based on the primary models unless otherwise stated. High LE was associated with a higher risk of depressive symptoms (AOR = 10.2; 95% CI: 8.0–12.9), while moderate LE was also significantly associated with depression (AOR = 3.1; 95% CI: 2.6–3.6).. To examine moderation effects, interaction terms were added in Models 1b, 2b, and 3b between LE and education, asset index, and rural-urban residence, respectively. In Model 1b, the interaction between LE and education was statistically significant, with lower odds of depression observed among individuals with secondary education experiencing moderate LE (AOR = 0.6; 95% CI: 0.5–0.8), individuals with college education experiencing moderate LE (AOR = 0.4; 95% CI: 0.2–0.9), and individuals with college education experiencing high LE (AOR = 0.4; 95% CI: 0.2–1.0). In model 2b, although asset index showed reduced odds ratios across LE categories, these interactions were not statistically significant. In Model 3b, the interaction between LE and rural-urban residence was also significant, with urban residents experiencing moderate LE having lower odds of depression compared to their rural counterparts (AOR = 0.7; 95% CI: 0.5–0.9).

**Table 2:** Results of logistic regression for the association between stressful life events and depression, adjusted for covariates.

	Variables	Model 1a	Model 1b	Model 2a	Model 2b	Model 3a	Model 3b
AOR (95%CI)	AOR (95%CI)	AOR (95%CI)	AOR (95%CI)	AOR (95%CI)	AOR (95%CI)
1	Life events (ref: Low)						
	Moderate	3.1 (2.6–3.6) ***	4.0 (3.2–5.0) ***	3.1 (2.6–3.6) ***	3.4 (2.6–4.5) ***	3.0 (2.6–3.6) ***	3.6 (2.9–4.5) ***
	High	10.2 (8.0–12.9) ***	12.4 (8.4–18.4) ***	9.8 (7.8–12.5) ***	12.3 (8.2–18.6) ***	9.8 (7.7–12.4) ***	9.9 (7.1–13.9) ***
2	Education (ref: Primary)						
	Secondary	0.8 (0.7–1.0) *	1.0 (0.8–1.2)				
	College	0.6 (0.5–0.9) *	1.1 (0.7–1.7)				
	Life event#Education						
	Moderate#Secondary		0.6 (0.5–0.8) ***				
	Moderate#College		0.4 (0.2–0.9) *				
	High#Secondary		0.7 (0.5–1.2)				
	High#College		0.4 (0.2–1.0) *				
3	Asset index (ref: Lower asset)						
	Higher asset			0.8 (0.7–0.9) *	0.9 (0.7–1.1)		
	Life event#Asset index						
	Moderate#Higher asset				0.8 (0.6–1.1)		
	High#Higher asset				0.7 (0.4–1.2)		
4	Residency (ref: Rural)						
	Urban					0.9 (0.8–1.1)	1.1 (0.9–1.3)
	Life event#Residency						
	Moderate#Urban						0.7 (0.5–0.9) *
	High#Urban						1.0 (0.6–1.5)
5	Age (ref 18–24 years)						
	25-34	0.7 (0.5–0.9) *	0.7 (0.5–0.9) *	0.7 (0.5–0.9) *	0.7 (0.5–0.9) *	0.7 (0.5–0.9) *	0.7 (0.5–0.9) *
	35-44	0.7 (0.5–0.9) *	0.7 (0.5–0.9) *	0.7 (0.5–1.0)	0.7 (0.5–1.0) *	0.7 (0.5–1.0) *	0.7 (0.5–1.0) *
	45-54	0.7 (0.5–1.0) *	0.7 (0.5–1.0) *	0.8 (0.6–1.1)	0.8 (0.6–1.1)	0.8 (0.6–1.1)	0.8 (0.6–1.1)
	55-64	0.9 (0.6–1.2)	0.9 (0.6–1.3)	1.0 (0.7–1.4)	1.0 (0.7–1.4)	1.0 (0.7–1.4)	1.05 (0.7–1.4)
	65-74	1.1 (0.7–1.5)	1.1 (0.7–1.6)	1.2 (0.8–1.7)	1.2 (0.8–1.7)	1.2 (0.9–1.7)	1.2 (0.9–1.8)
	≥75	1.7 (1.1–2.8) **	1.8 (1.2–2.9) *	2.0 (1.3–3.1) **	2.0 (1.3–3.1) **	2.0 (1.3–3.2) **	2.1 (1.3–3.2) **
6	Sex (ref male)						
	Female	1.3 (1.1–1.6) ***	1.3 (1.1–1.6) ***	1.3 (1.2–1.6) ***	1.3 (1.2–1.6) ***	1.3 (1.1–1.6) ***	1.3 (1.1–1.6) ***
7	Marital status (ref: Single)						
	Married	0.5 (0.4–0.6) ***	0.5 (0.4–0.6) ***	0.5 (0.4–0.6) ***	0.5 (0.4–0.6) ***	0.5 (0.4–0.6) ***	0.5 (0.4–0.6) ***
	Widowed	0.7 (0.5–1.0) *	0.7 (0.5–1.0) *	0.7 (0.5–1.0)	0.7 (0.5–1.0)	0.7 (0.5–1.0)	0.7 (0.5–1.0)
	Divorced	0.6 (0.4–1.0)	0.6 (0.4–1.1)	0.6 (0.4–1.0)	0.6 (0.4–1.0)	0.6 (0.4–1.1)	0.6 (0.4–1.1)
8	Province (ref: West Java)						
	Central Java	1.6 (1.3–2.0) ***	1.6 (1.3–2.0) ***	1.4 (1.2–1.8) ***	1.4 (1.2–1.8) **	1.5 (1.2–1.9) ***	1.5 (1.2–1.8) **
	East Java	1.7 (1.4–2.1) ***	1.7 (1.4–2.1) ***	1.6 (1.3–2.0) ***	1.6 (1.3–2.0) ***	1.6 (1.3–2.0) ***	0.7 (0.5–1.0) ***
	Banten	0.8 (0.6–1.2)	0.8 (0.5–1.2)	0.7 (0.5–1.1)	0.7 (0.5–1.1)	0.7 (0.5–1.1)	0.6 (0.4–1.1)
	Log Likelihood	−3161.9	−3155.7	−3163.2	−3162.0	−3165.9	−3163.1
	Nagelkerke Pseudo-R2	0.101	0.103	0.100	0.101	0.099	0.077
	Hosmer-Lemeshow	8.3	9.2	7.8	11.3	14.0	10.2
	Chi2	0.4	0.3	0.4	0.2	0.1	0.2
	Observations	19,113	19,113	19,122	19,122	19,122	19,122

Note: AOR = Adjusted Odds Ratio; CI  = Confidence Interval in parenthesis; ***p < 0.001, **p < 0.005, * p < 0.05. Model a: without interaction, Model b: with interaction.

**Table 3:** Results of logistic regression for the association between stressful life events and anxiety, adjusted with covariates.

	Variables	Model 1a	Model 1b	Model 2a	Model 2b	Model 3a	Model 3b
AOR (95%CI)	AOR (95%CI)	AOR (95%CI)	AOR (95%CI)	AOR (95%CI)	AOR (95%CI)
1	Life events (ref: Low)						
	Moderate	2.7 (2.5–3.1) ***	3.2 (2.7–3.8) ***	2.7 (2.4–3.0) ***	3.3 (2.7–3.9) ***	2.7 (2.4–3.0) ***	3.2 (2.7–3.7) ***
	High	6.5 (5.3–8.0) ***	7.7 (5.5–11.0) ***	6.2 (5.1–7.6) ***	7.2 (4.9–10.4) ***	6.3 (5.2–7.7) ***	6.7 (5.0–8.9) ***
2	Education (ref: Primary)						
	Secondary	0.8 (0.7–0.9) ***	0.9 (0.7–1.0) *				
	College	0.6 (0.5–0.8) ***	0.8 (0.6–1.2)				
	Life event#Education						
	Moderate#Secondary		0.8 (0.6–0.9) *				
	Moderate#College		0.5 (0.3–0.8) *				
	High#Secondary		0.7 (0.4–1.1)				
	Severe#College		0.9 (0.5–1.8)				
3	Asset index (ref: Lower asset)						
	Higher asset			0.9 (0.8–1.1)*	1.0 (0.8–1.2)		
	Life event#Asset index						
	Moderate#Higher asset				0.8 (0.6–1.0)*		
	High#Higher asset				0.8 (0.5–1.2)		
4	Residency (ref: Rural)						
	Urban					0.8 (0.7–0.9) ***	0.9 (0.7–1.0)
	Life event#Residency						
	Moderate#Urban						0.7 (0.6–0.9) *
	High#Urban						0.6 (0.5–1.3)
5	Age (ref 18–24 years)						
	25-34	0.8 (0.7–1.0)	0.8 (0.7–1.0) *	0.8 (0.7–1.0)	0.8 (0.7–1.0)	0.8 (0.7–1.0)	0.8 (0.7–1.0)
	35-44	0.8 (0.7–1.0)	0.8 (0.6–1.0)	0.9 (0.7–1.1)	0.9 (0.7–1.1)	0.9 (0.7–1.1)	0.9 (0.7–1.1)
	45-54	0.8 (0.6–0.9)	0.8 (0.6–0.9) *	0.8 (0.7–1.0)	0.8 (0.7–1.0)	0.8 (0.7–1.0)	0.8 (0.7–1.0)
	55-64	0.7 (0.6–0.9)	0.7 (0.6–0.9) *	0.8 (0.6–1.0)	0.8 (0.6–1.0)	0.8 (0.6–1.0)	0.8 (0.7–1.0)
	65-74	0.9 (0.7–1.2)	0.9 (0.7–1.2)	1.0 (0.8–1.4)	1.0 (0.8–1.4)	1.0 (0.8–1.4)	1.0 (0.8–1.4)
	≥75	0.9 (0.6–1.3)	0.9 (0.6–1.4)	1.1 (0.7–1.6)	1.1 (0.7–1.6)	1.0 (0.7–1.5)	1.1 (0.7–1.6)
6	Sex (ref male)						
	Female	1.5 (1.3–1.6) ***	1.5 (1.3–1.6) ***	1.5 (1.3–1.6) ***	1.5 (1.3–1.7) ***	1.5 (1.3–1.7) ***	1.5 (1.3–1.7) ***
7	Marital status (ref: Single)						
	Married	0.6 (0.5–0.8) ***	0.7 (0.5–0.8) ***	0.6 (0.5–0.8) ***	0.7 (0.5–0.8) ***	0.6 (0.5–0.8) ***	0.6 (0.5–0.8) ***
	Widowed	0.7 (0.5–0.9) *	0.7 (0.5–0.9) *	0.7 (0.5–0.9) *	0.7 (0.5–0.9) *	0.7 (0.5–0.9) *	0.7 (0.5–0.9) *
	Divorced	0.9 (0.6–1.3)	0.9 (0.6–1.3)	0.9 (0.6–1.3)	0.9 (0.6–1.3)	0.9 (0.6–1.3)	0.9 (0.6–1.3)
8	Province (ref: West Java)						
	Central Java	1.2 (1.0–1.4) **	1.2 (1.0–1.4) *	1.1 (1.0–1.3)	1.1 (1.0–1.3)	1.2 (1.0–1.4) *	1.2 (1.0–1.4) *
	East Java	1.0 (0.9–1.2)	1.0 (0.9–1.2)	1.0 (0.8–1.1)	1.0 (0.8–1.1)	1.0 (0.8–1.0)	0.9 (0.8–1.1)
	Banten	1.0 (0.9–1.3)	1.0 (0.8–1.2)	0.9 (0.7–1.1)	0.9 (0.7–1.1)	1.0 (0.8–1.3)	1.0 (0.8–1.3)
	Log Likelihood	−5268.6	−3917.9	−5276.3	−5273.6	−5269.1	−5265.4
	Nagelkerke Pseudo-R2	0.069	0.070	0.067	0.068	0.069	0.070
	AIC	0.54	0.55	0.55	0.55	0.55	0.55
	Hosmer-Lemeshow Chi2	3.1	10.0	4.6	3.9	23.0	20.6
	Observations	19,123	19,123	19,132	19,132	19,132	19,132

Note: AOR = Adjusted Odds Ratio; CI  = Confidence Interval in parenthesis; ***p < 0.001, **p < 0.005, * p < 0.05. Model a: without interaction, Model b: with interaction.

**Fig 2:**
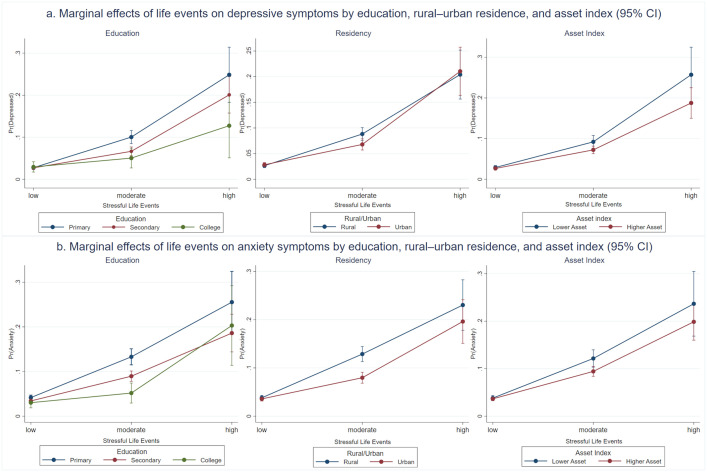
Marginal effects of life events on mental health outcomes, adjusted for age and sex: (a) depressive symptoms by education, rural–urban residence, and asset index; (b) anxiety symptoms by education, rural–urban residence, and asset index.

Similarly, both moderate and high LE were significantly associated with higher odds of anxiety (AOR = 2.7, 95% CI: 2.5–3.1; AOR = 6.5, 95% CI: 5.3–8.0, respectively). In Model 1b, the interaction between LE and educational attainment was significant, with lower odds of anxiety observed among individuals with college education (AOR = 0.5; 95% CI: 0.3–0.8) and secondary education (AOR = 0.8; 95% CI: 0.6–0.9) experiencing moderate LE. In Model 2b, although a higher asset index was associated with reduced odds of anxiety among participants with moderate LE (AOR = 0.8; 95% CI: 0.6–1.0), this interaction did not reach statistical significance. In Model 3b, urban residents experiencing moderate LE had lower odds of anxiety compared to their rural counterparts (AOR = 0.7; 95% CI: 0.6–0.9). Fig 2 further clarify these interaction effects between life events, education, asset index, and rural-urban residency. Detailed frequencies of the various life events experienced by respondents are provided in S2 Table. To test the robustness of our findings, we repeated the analyses using multiple imputation (S3a and S3b Tables). In comparison with the multiple imputation approach, the complete case analysis produced wider standard errors, but the overall results remained consistent. The lack of meaningful differences between the two approaches supports the robustness of our conclusions.

## Discussion

This study aimed to examine the associations between life events (LE), depression, and anxiety among adults in Java, Indonesia, and to assess how these associations vary by education, household wealth, and place of residence. By using a large population-based dataset and explicitly testing socioeconomic moderation, this study extends existing evidence from LMIC settings, where such analyses remain limited.

### Study aim 1

In our sample, 4.4% of participants reported depressive symptoms and 8.5% reported anxiety symptoms. Those exposed to high levels of life events had markedly elevated risks, with more than tenfold higher odds of depression (AOR = 10.2, 95% CI: 8.0–12.9) and over sevenfold higher odds of anxiety (AOR = 6.5, 95% CI: 5.3–8.0). Supporting this result, a study conducted in Hong Kong among older adults found that the death of a spouse, a life event with the highest Life Change Unit (LCU) score according to the Holmes-Rahe Stress Inventory [55], was significantly associated with increased depressive symptoms [12]. This association underscores the profound psychological impact of major life stressors. This finding is consistent with evidence showing that events involving threats, illness, conflicts or bereavement are especially influential in triggering or intensifying anxiety symptoms [34,69]. These patterns can be better understood through the stress-diathesis framework, which posits that life events act as external stressors that may precipitate the onset of depression or anxiety [70].

Although research consistently shows that stressful experiences play a significant role in triggering depressive episodes [71,72], not everyone exposed to stress develops depression. This variation is explained by the presence of a diathesis, biological, psychological, or social, that shapes an individual’s vulnerability to the effects of stress [70]. Besides that, the interplay between life events and individual psychological resilience, characterised by factors such as personality [73] and coping skills [34], can mediate or exacerbate the effects of these events on mental health. For instance, a large family-based study using polygenic risk scores for major depressive disorder demonstrated that genetic vulnerability significantly interacted with stressful life events to increase depression risk, supporting the multiplicative diathesis–stress model [74]. These findings underscore that depression is not merely the result of stress exposure, but emerges when environmental stressors interact with underlying vulnerabilities. Moreover, exposure to such events may not only initiate mental health disturbances but also worsen or prolong the course of existing depression or anxiety disorders [13].

### Study aim 2

Under the second study aim, we examined whether the associations between life events and mental health outcomes varied across socioeconomic contexts, focusing on asset index, education, and rural–urban residence.

We found that a higher asset index appeared to be a protective factor against anxiety among individuals experiencing moderate life events. However, it showed no significant association with depression. This finding is consistent with a study using the German National Health Interview, which observed that while higher household income was initially associated with fewer mental health problems in children and adolescents, the effect became non-significant when the number of stressful life situations was accounted for [75], a finding that suggests that life stressors, rather than income alone, play a more direct role in mental health outcomes [75]. A similar pattern was observed in a U.S.-based study, where adults with fewer financial assets (less than $5,000) were found to have more than twice the odds of screening positive for depression, anxiety, or both, compared to those with financial assets of $100,000 or more [76]. Interestingly, annual household income in that study was not significantly associated with anxiety symptoms after adjusting for asset levels, underscoring that the stability and accessibility of financial resources may be more critical than income per se in buffering psychological distress. These findings support the broader notion that individuals with greater financial means are generally better equipped to manage stress through improved access to healthcare, social support, and other coping mechanisms [44]. In LMICs, where structural inequalities are more pronounced, low socioeconomic status may further exacerbate the negative impact of life events, increasing vulnerability to mental health disorders. Taken together, the protective effect of socioeconomic resources is context-dependent and shaped by their quality, stability, and accessibility. In Indonesia, asset may also shape disparities in healthcare access following life events, as households with greater resources are better positioned to seek timely care. Similar inequities have been observed in conflict-affected regions of Nigeria, where Musa et al. reported that structural barriers and resource disparities exacerbate mental health consequences [77]. These comparisons highlight the importance of broader structural determinants in shaping mental health outcomes.

We further found that education moderated the associations between LE and the odds of depressive and anxiety symptoms (Fig 2). Specifically, individuals with secondary or higher education experiencing moderate LE, and those with a college education experiencing high LE, had lower odds of reporting depressive symptoms. This finding aligns with a study using the Danish National Health Survey 2010, which also emphasised the protective role of education, showing that individuals with higher education were far less likely to experience negative social life events (e.g., only around 2–3% among those with higher education compared with more than half among those with basic education) [78].

Several plausible mechanisms explain how education may buffer the psychological impact of stressful life events. First, higher educational attainment is associated with enhanced coping skills, greater resilience, and improved access to economic stability, employment opportunities, and health information, all of which may mitigate the mental health impacts of stressful life events [17,18]. This protective role of education appears more pronounced for depression than for anxiety, possibly because anxiety more strongly influences educational outcomes, such as dropout rates and self-concept regarding education, rather than being alleviated by them [79]. Second, individuals with higher education tend to possess greater capacity to cope with stressful life events, making them better equipped to navigate stress and, consequently, less likely to experience adverse effects from such events [43]. Education also acts as a protective factor by improving access to health information and expanding economic opportunities. For example, individuals with higher educational attainment are more likely to secure stable and well-paying employment [80].

The observed moderating role of urban–rural residence can be understood in light of differences in social connectivity, service availability, and environmental exposures.. Urban residence is associated with lower odds of depression and anxiety among those experiencing moderate LE. One plausible explanation is that urban environments often facilitate social connectivity and access to mental health resources [24]. Increased population density typically leads to more varied opportunities for social interactions, which can foster interpersonal relationships essential during stressful life events [81]. On the contrary, living in urban areas is correlated with increased mental health problems due to air pollution, noise, and urban greenness [24,80]. However, in developed countries, living in urban areas correlates with the risk of loneliness and depression, especially in older people [45].

### Role of individual demographic characteristics in mental health

Our study also found that some demographic covariates were associated with depression and anxiety symptoms. We found that being female was associated with higher odds of depressive and anxiety symptoms, which supports prior studies revealing that females are more vulnerable to depression [82]. Several factors may explain this vulnerability, including hormonal fluctuations during puberty, pregnancy, and menopause; greater exposure to interpersonal stress; and a higher likelihood of experiencing atypical depressive symptoms and comorbid anxiety disorders [83,84]. Our study found that being married offered protection against both depression and anxiety. This aligns with evidence that marriage can lessen psychological distress [85]. In terms of age, this study found that individuals aged 25–34 were less likely to experience depression, while those aged 75 and older were more likely to do so. The major factors contributing to depression among older individuals included lack of family support, feelings of unworthiness and being unwanted, economic dependency, poor physical health, and other family-related issues. These findings highlight the crucial role that family plays in maintaining the mental well-being of the elderly [86].

The observed regional differences in depressive and anxiety symptoms may partly reflect underlying socioeconomic and contextual variation across provinces in Java. Compared with West Java, Central Java has a higher poverty rate, lower minimum wages, and lower literacy development scores [87], factors that may increase vulnerability to psychological distress. Central Java also exhibits lower labour market participation and lower household economic indicators, which may compound exposure to stressors. In contrast, East Java, while also characterised by relatively high poverty prevalence, shows higher literacy and happiness indices, which may offer some protective effects despite economic constraints. These contextual differences, summarised in S4 Table, provide a plausible background for the elevated odds of anxiety observed in Central Java and the heterogeneous patterns of depressive symptoms across provinces.

### Strengths and limitations

This study has strengths, including a large sample size of 19,186 individuals, conducted across five provinces on the island of Java, which helps represent the mental health conditions related to LE experienced by the population over the past year.

Nevertheless, this study has several limitations. First, its cross-sectional design precludes causal inference; the observed associations between life events and mental health outcomes are correlational and may be bidirectional. Second, the LE measures relied on self-reported recall over the past year, potentially introducing recall bias. Respondents might underreport or overreport experiences depending on their current psychological state, potentially influencing the accuracy of the associations observed. Second, although the SRRS was translated into Indonesian and reviewed for conceptual equivalence [88], it has not been formally validated in the Indonesian context, as typically done in research [89]. As such, the scale may not fully capture culturally specific stressors or life events that are salient in Indonesia, such as extended family obligations, communal responsibilities, or local socio-economic challenges. Future studies could benefit from longitudinal designs and objective measures or repeated assessments of life events based on the sociocultural landscape to strengthen causal inference. Third, although multiple imputation was employed to handle missing data, this approach relies on the missing-at-random (MAR) assumption. If data are missing not at random, the imputation model may not fully account for the missing-data mechanism, potentially introducing bias. To minimise this risk, a comprehensive set of auxiliary variables was included in the imputation model. Results from both the imputed and complete case analyses are provided in the supplementary tables, and their consistency suggests that multiple imputation did not substantially bias the study findings. Finally, it has not measured other variables which may be associated with mental health, such as genetic factors [90], population density [91], personality traits [73], psychological factors include postpartum conditions [92], or events related to climate change [93–95]. Future research could address these diverse influences by including these variables to understand the complex determinants of mental health more comprehensively.

## Conclusion

In conclusion, life events are associated with depression and anxiety symptoms among adults in Java, Indonesia, with lower risks observed among individuals with higher education and those living in urban areas. These findings highlight the importance of socioeconomic and residential context in shaping vulnerability to stress-related mental health outcomes. From a health policy development perspective, mental health strategies should prioritise populations exposed to repeated life stressors and structural disadvantage, particularly those with lower educational attainment and living in rural settings. Integrating psychosocial support within community-based and primary care services may help mitigate the mental health impact of life events and reduce existing inequalities.

## Supporting information

S1 Appendix Stress-diathesis model. (DOCX)

S2 Table Frequency of respondents who answered “YES” to the items on the Social Readjustment Rating Scale by Holmes and Rahe. (DOCX)

S3a Table Results of logistic regression for the association between stressful life events and depression, adjusted for covariates. (DOCX)

S3b Table Results of logistic regression for the association between stressful life events and anxiety, adjusted with covariates. (DOCX)

S4 Table Descriptive information of each province. (DOCX)
