# Supplementary material for: The association between life events and mental health among adults in Java, Indonesia: Investigating the moderating effects by education, asset index, and rural-urban area of residence
Source: PLoS One. 2026 May 18;21(5):e0348726. doi: 10.1371/journal.pone.0348726 (PMC13183217; doi:10.1371/journal.pone.0348726)
Supplement: S1 Appendix — (DOCX) [file pone.0348726.s001.docx]

**S1 Appendix. Stress-diathesis model**

The stress–diathesis model is a foundational theoretical framework for understanding the aetiology of common mental disorders, particularly depression and anxiety. The model posits that mental disorders do not arise solely from exposure to stressful life events, but rather from the interaction between environmental stressors and an individual’s underlying vulnerability, or diathesis. Diathesis refers to a latent predisposition that may be biological (e.g., genetic liability, neurobiological sensitivity), psychological (e.g., personality traits, maladaptive cognitive styles), or social (e.g., early-life adversity, chronic socioeconomic disadvantage). This vulnerability may remain dormant unless activated by stressors of sufficient type or magnitude.

Monroe and Simons (1991) proposed three models of the stress–diathesis relationship, which differ in the assumed causal roles of stress and diathesis. These models provide important insights into why exposure to similar life events can result in markedly different mental health outcomes across individuals.

**Model 1: Stress-Activated Diathesis (Selective Stress Model)**

In the first model, diathesis is conceptualised as a latent vulnerability that becomes activated only in the presence of specific stressors. Stressors are classified into two types: Stress1, related to the individual’s underlying diathesis, and Stress2, unrelated to that vulnerability. According to this model, not all stressful life events have equal pathogenic potential. Depression or anxiety emerges when a person with an underlying diathesis encounters stressors that are congruent with, or specifically activate, that vulnerability.

Under this framework, mental disorders arise from “the right kind of stress in a vulnerable individual.” For example, interpersonal loss may be particularly led into depression for individuals with attachment-related vulnerabilities, whereas occupational stress may be more salient for individuals whose self-worth is strongly tied to work performance. Individuals without the relevant diathesis may experience the same stressors without developing psychopathology. This model, therefore, emphasises stress specificity and explains substantial heterogeneity in individual responses to life events.

Model1 . Stress-Activated Diathesis (Selective Stress Model)

**Model 2: Diathesis-Driven Stress Generation (Diathesis as Primary Cause)**

The second model places diathesis as the primary causal factor, with stress playing a secondary or consequential role. In this formulation, individuals with a strong underlying vulnerability are more likely to experience stressful life events due to their predisposition. Stress is not viewed as an independent causal trigger but rather as an outcome or manifestation of the diathesis itself.

For instance, individuals with depressive cognitive styles, emotional dysregulation, or maladaptive interpersonal behaviours may inadvertently generate stressful environments, such as relationship conflict, job instability, or social isolation. These stressors then reinforce or exacerbate existing symptoms, creating a self-perpetuating cycle. In this model, the apparent association between life events and mental disorders may reflect stress generation rather than stress causation. This perspective is particularly relevant for understanding recurrent depression, where individuals with established vulnerability may repeatedly encounter adverse life circumstances partly shaped by their own behaviours, coping styles, or social contexts.

Model2. Diathesis-Driven Stress Generation (Diathesis as Primary Cause)

**Model 3: Stress-Dominant Model (Stress as Primary Cause)**

The third model conceptualises stress as the primary causal factor in the development of mental disorders, while diathesis plays a minimal or non-essential role. Similar to Model 1, stressors are divided into Stress1 (related to diathesis) and Stress2 (unrelated to diathesis), but unlike Model 1, vulnerability is not required for psychopathology to emerge. Instead, sufficiently severe or cumulative stress exposure can precipitate depression or anxiety in most individuals, regardless of pre-existing vulnerability.

This model is often invoked in contexts of extreme or widespread stress, such as natural disasters, armed conflict, economic crises, or major bereavement. In such situations, the magnitude or chronicity of stress may overwhelm individual coping resources, leading to elevated population-level prevalence of mental disorders. Here, diathesis may modify severity or recovery trajectories but is not necessary for onset.

Model3. Stress-Dominant Model (Stress as Primary Cause)

Together, these three models highlight that the relationship between life events and mental health is neither uniform nor unidirectional. Stressful life events may act as triggers, consequences, or primary causes of depression and anxiety, depending on the presence and nature of individual vulnerabilities and contextual factors. Importantly, these models are not mutually exclusive and may operate simultaneously or sequentially across the life course.

In population-based studies, such as the present analysis, the stress–diathesis framework provides a conceptual basis for examining how exposure to life events interacts with socioeconomic conditions, such as education, wealth, and place of residence, to shape mental health outcomes. Socioeconomic disadvantage may function both as a chronic stressor and as a source of diathesis, increasing vulnerability to the psychological impact of acute life events.

Source: Monroe SM, Simons AD. Diathesis-stress theories in the context of life stress research: implications for the depressive disorders. Psychol Bull. 1991 Vol 110(3):406-25. doi: 10.1037/0033-2909.110.3.406. PMID: 1758917.
