# Supplementary material for: The association between life events and mental health among adults in Java, Indonesia: Investigating the moderating effects by education, asset index, and rural-urban area of residence
Source: PLoS One. 2026 May 18;21(5):e0348726. doi: 10.1371/journal.pone.0348726 (PMC13183217; doi:10.1371/journal.pone.0348726)
Supplement: S2 Table — (DOCX) [file pone.0348726.s002.docx]

**S2 Table.** **Frequency of respondents who answered "YES" to the items on the Social Readjustment Rating Scale by Holmes and Rahe.**

|  | **Answered yes to the question** | **n** | **%** | **missing** |
| --- | --- | --- | --- | --- |
| 1 | Death of spouse? | 229 | 1.19 | 13 (0.07) |
| 2 | Divorce | 149 | 0.78 | 24 (0.13) |
| 3 | Marital Separation from mate? | 907 | 4.73 | 15 (0.08) |
| 4 | Detention in jail or other institution? | 40 | 0.21 | 32 (0.17) |
| 5 | Death of a close family member? | 4,280 | 22.31 | 17 (0.09) |
| 6 | Major personal injury or illness? | 1,236 | 6.44 | 17 (0.09) |
| 7 | Marriage | 1,055 | 5.50 | 21 (0.11) |
| 8 | Being fired at work? | 126 | 0.66 | 20 (0.10) |
| 9 | Marital reconciliation with mate? | 49 | 0.26 | 29 (0.15) |
| 10 | Retirement from work? | 124 | 0.65 | 24 (0.13) |
| 11 | Major change in the health or behaviour of a family member? | 1,543 | 8.04 | 16 (0.08) |
| 12 | Pregnancy | 543 | 2.83 | 23 (0.12) |
| 13 | Sexual Difficulties? | 273 | 1.42 | 31 (0.16) |
| 14 | Gaining a new family member (i.e. . birth, adoption, older adult moving in) | 1,946 | 10.14 | 23 (0.12) |
| 15 | Major business readjustment? | 919 | 4.79 | 32 (0.17) |
| 16 | Major change in financial state (i.e. ... a lot worse or better off than usual)? | 6,479 | 33.77 | 18 (0.09) |
| 17 | Death of a close friend? | 1,546 | 8.06 | 21 (0.11) |
| 18 | Changing to a different line of work? | 713 | 3.72 | 33 (0.17) |
| 19 | Major change in the number of arguments w/spouse (a lot more or a lot less)? | 1,438 | 7.50 | 21 (0.11) |
| 20 | Taking on a mortgage (for home, business, etc.)? | 2,854 | 14.88 | 18 (0.09) |
| 21 | Foreclosure on a mortgage or loan? | 75 | 0.39 | 18 (0.09) |
| 22 | Major change in responsibilities at work (i.e. promotion, demotion, etc.)? | 381 | 1.99 | 18 (0.09) |
| 23 | Son or daughter leaving home (marriage, attending college, joined military)? | 1,339 | 6.98 | 19 (0.10) |
| 24 | In-law troubles? | 118 | 0.62 | 13 (0.07) |
| 25 | Outstanding personal achievement? | 1,394 | 7.27 | 19 (0.10) |
| 26 | Spouse beginning or ceasing work outside the home? | 219 | 1.14 | 19 (0.10) |
| 27 | Beginning or ceasing formal schooling? | 252 | 1.31 | 23 (0.12) |
| 28 | Major change in living condition (new home, remodelling, deterioration neighbour | 1,806 | 9.41 | 26 (0.14) |
| 29 | Revision of personal habits (dress manners, associations, quitting smoking)? | 1,597 | 8.32 | 21 (0.11) |
| 30 | Troubles with the boss? | 179 | 0.93 | 25 (0.13) |
| 31 | Major changes in working hours or conditions? | 1,105 | 5.76 | 22 (0.11) |
| 32 | Changes in residence? | 375 | 1.95 | 14 (0.07) |
| 33 | Changing to a new school? | 39 | 0.20 | 35 (0.18) |
| 34 | Major change in usual type and/or amount of recreation? | 2,434 | 12.69 | 22 (0.11) |
| 35 | Major change in religious activity (i.e. ... a lot more or less than usual) | 4,564 | 23.79 | 18 (0.09) |
| 36 | Major change in social activities (clubs, movies, visiting, etc.) | 2,744 | 14.30 | 22 (0.11) |
| 37 | Taking on a loan (such as to buy a car, motorcycle, phone, etc.) | 3,958 | 20.63 | 23 (0.12) |
| 38 | Major change in sleeping habits (a lot more or a lot less than usual) | 866 | 4.51 | 22 (0.11) |
| 39 | Major change in number of family get-togethers | 2,458 | 12.81 | 22 (0.11) |
| 40 | Major change in eating habits (a lot more or less food intake, or very different ) | 3,948 | 20.58 | 19 (0.10) |
| 41 | Vacation | 7,105 | 37.03 | 18 (0.09) |
| 42 | Major holidays (Eid al-Fitr, Christmas, etc.) | 16,685 | 86.96 | 10 (0.05) |
| 43 | Minor violations of the law (traffic tickets, fine, disturbing the peace, etc). | 541 | 2.82 | 12 (0.06) |
