# Supplementary material for: The association between life events and mental health among adults in Java, Indonesia: Investigating the moderating effects by education, asset index, and rural-urban area of residence
Source: PLoS One. 2026 May 18;21(5):e0348726. doi: 10.1371/journal.pone.0348726 (PMC13183217; doi:10.1371/journal.pone.0348726)
Supplement: S3a Table — (DOCX) [file pone.0348726.s003.docx]

**S3a Table. Results of logistic regression for the association between stressful life events and depression, adjusted for covariates.**

|  | **Variables** | **Model 1a** | **Model 1b** | **Model 2a** | **Model 2b** | **Model 3a** | **Model 3b** |
| --- | --- | --- | --- | --- | --- | --- | --- |
|  |  | AOR (95%CI) | AOR (95%CI) | AOR (95%CI) | AOR (95%CI) | AOR (95%CI) | AOR (95%CI) |
| 1 | Life events (ref: Low) |  |  |  |  |  |  |
|  | Moderate | 3.1 (2.6 – 3.6) *** | 4.0 (3.2 – 5.0) *** | 3.1 (2.6 – 3.6) *** | 3.4 (2.6 – 4.5) *** | 3.0 (2.6 – 3.6) *** | 3.6 (2.9 – 4.5) *** |
|  | High | 10.2 (8.0 – 12.9) *** | 12.4 (8.4 – 18.4) *** | 9.8 (7.8 – 12.5) *** | 12.3 (8.2 – 18.6) *** | 9.8 (7.7 – 12.4) *** | 9.9 (7.1 – 13.9) *** |
| 2 | Education (ref: Primary) |  |  |  |  |  |  |
|  | Secondary | 0.8 (0.7 – 1.0) * | 1.0 (0.8 – 1.2) |  |  |  |  |
|  | College | 0.6 (0.5 – 0.9) * | 1.1 (0.7 – 1.7) |  |  |  |  |
|  | Life event#Education |  |  |  |  |  |  |
|  | Moderate#Secondary |  | 0.6 (0.5 – 0.8) *** |  |  |  |  |
|  | Moderate#College |  | 0.4 (0.2 – 0.9) * |  |  |  |  |
|  | High#Secondary |  | 0.7 (0.5 – 1.2) |  |  |  |  |
|  | High#College |  | 0.4 (0.2 – 1.0) * |  |  |  |  |
| 3 | Asset index  (ref: Lower asset) |  |  |  |  |  |  |
|  | Higher asset |  |  | 0.8 (0.7 – 0.9) * | 0.9 (0.7 – 1.1) |  |  |
|  | Life event#Asset index |  |  |  |  |  |  |
|  | Moderate#Higher asset |  |  |  | 0.8 (0.6 – 1.1) |  |  |
|  | High#Higher asset |  |  |  | 0.7 (0.4 – 1.2) |  |  |
| 4 | Residency (ref: Rural) |  |  |  |  |  |  |
|  | Urban |  |  |  |  | 0.9 (0.8 – 1.1) | 1.1 (0.9 – 1.3) |
|  | Life event#Residency |  |  |  |  |  |  |
|  | Moderate#Urban |  |  |  |  |  | 0.7 (0.5 – 0.9) * |
|  | High#Urban |  |  |  |  |  | 1.0 (0.6 – 1.5) |
| 5 | Age (ref 18-24 years) |  |  |  |  |  |  |
|  | 25-34 | 0.7 (0.5 – 0.9) * | 0.7 (0.5 – 0.9) * | 0.7 (0.5 – 0.9) * | 0.7 (0.5 – 0.9) * | 0.7 (0.5 – 0.9) * | 0.7 (0.5 – 0.9) * |
|  | 35-44 | 0.7 (0.5 – 0.9) * | 0.7 (0.5 – 0.9) * | 0.7 (0.5 – 1.0) | 0.7 (0.5 – 1.0) * | 0.7 (0.5 – 1.0) * | 0.7 (0.5 – 1.0) * |
|  | 45-54 | 0.7 (0.5 – 1.0) * | 0.7 (0.5 – 1.0) * | 0.8 (0.6 – 1.1) | 0.8 (0.6 – 1.1) | 0.8 (0.6 – 1.1) | 0.8 (0.6 – 1.1) |
|  | 55-64 | 0.9 (0.6 – 1.2) | 0.9 (0.6 – 1.3) | 1.0 (0.7 – 1.4) | 1.0 (0.7 – 1.4) | 1.0 (0.7 – 1.4) | 1.05 (0.7 – 1.4) |
|  | 65-74 | 1.1 (0.7 – 1.5) | 1.1 (0.7 – 1.6) | 1.2 (0.8 – 1.7) | 1.2 (0.8 – 1.7) | 1.2 (0.9 – 1.7) | 1.2 (0.9 – 1.8) |
|  | ≥75 | 1.7 (1.1 – 2.8) ** | 1.8 (1.2 – 2.9) * | 2.0 (1.3 – 3.1) ** | 2.0 (1.3 – 3.1) ** | 2.0 (1.3 – 3.2) ** | 2.1 (1.3 – 3.2) ** |
| 6 | Sex (ref male) |  |  |  |  |  |  |
|  | Female | 1.3 (1.1 – 1.6) *** | 1.3 (1.1 – 1.6) *** | 1.3 (1.2 – 1.6) *** | 1.3 (1.2 – 1.6) *** | 1.3 (1.1 – 1.6) *** | 1.3 (1.1 – 1.6) *** |
| 7 | Marital status (ref: Single) |  |  |  |  |  |  |
|  | Married | 0.5 (0.4 – 0.6) *** | 0.5 (0.4 – 0.6) *** | 0.5 (0.4 – 0.6) *** | 0.5 (0.4 – 0.6) *** | 0.5 (0.4 – 0.6) *** | 0.5 (0.4 – 0.6) *** |
|  | Widowed | 0.7 (0.5 – 1.0) * | 0.7 (0.5 – 1.0) * | 0.7 (0.5 – 1.0) | 0.7 (0.5 – 1.0) | 0.7 (0.5 – 1.0) | 0.7 (0.5 – 1.0) |
|  | Divorced | 0.6 (0.4 – 1.0) | 0.6 (0.4 – 1.1) | 0.6 (0.4 – 1.0) | 0.6 (0.4 – 1.0) | 0.6 (0.4 – 1.1) | 0.6 (0.4 – 1.1) |
| 8 | Province (ref: West Java) |  |  |  |  |  |  |
|  | Central Java | 1.6 (1.3 – 2.0) *** | 1.6 (1.3 – 2.0) *** | 1.4 (1.2 – 1.8) *** | 1.4 (1.2 – 1.8) ** | 1.5 (1.2 – 1.9) *** | 1.5 (1.2 – 1.8) ** |
|  | East Java | 1.7 (1.4 – 2.1) *** | 1.7 (1.4 – 2.1) *** | 1.6 (1.3 – 2.0) *** | 1.6 (1.3 – 2.0) *** | 1.6 (1.3 – 2.0) *** | 0.7 (0.5 – 1.0) *** |
|  | Banten | 0.8 (0.6 – 1.2) | 0.8 (0.5 – 1.2) | 0.7 (0.5 – 1.1) | 0.7 (0.5 – 1.1) | 0.7 (0.5 – 1.1) | 0.6 (0.4 – 1.1) |
|  | Log Likelihood | -3161.9 | -3155.7 | -3163.2 | -3162.0 | -3165.9 | -3163.1 |
|  | Nagelkerke Pseudo-R2 | 0.101 | 0.103 | 0.100 | 0.101 | 0.099 | 0.077 |
|  | Hosmer-Lemeshow | 8.3 | 9.2 | 7.8 | 11.3 | 14.0 | 10.2 |
|  | Chi2 | 0.4 | 0.3 | 0.4 | 0.2 | 0.1 | 0.2 |
|  | Observations | 19,113 | 19,113 | 19,122 | 19,122 | 19,122 | 19,122 |

Note: AOR=Adjusted Odds Ratio; CI =Confidence Interval in parenthesis; ***p < 0.001, **p < 0.005, * p < 0.05. Model a: without interaction, Model b: with interaction
