# Supplementary material for: The association between life events and mental health among adults in Java, Indonesia: Investigating the moderating effects by education, asset index, and rural-urban area of residence
Source: PLoS One. 2026 May 18;21(5):e0348726. doi: 10.1371/journal.pone.0348726 (PMC13183217; doi:10.1371/journal.pone.0348726)
Supplement: S3b Table — (DOCX) [file pone.0348726.s004.docx]

**Supplementary Table 3b. Results of logistic regression for the association between stressful life events and anxiety, adjusted with covariates.**

|  | **Variables** | **Model 1a** | **Model 1b** | **Model 2a** | **Model 2b** | **Model 3a** | **Model 3b** |
| --- | --- | --- | --- | --- | --- | --- | --- |
|  |  | AOR (95%CI) | AOR (95%CI) | AOR (95%CI) | AOR (95%CI) | AOR (95%CI) | AOR (95%CI) |
| 1 | Life events (ref: Low) |  |  |  |  |  |  |
|  | Moderate | 2.7 (2.5 – 3.1) *** | 3.2 (2.7– 3.8) *** | 2.7 (2.4 – 3.0) *** | 3.3 (2.7 – 3.9) *** | 2.7 (2.4 – 3.0) *** | 3.2 (2.7– 3.7) *** |
|  | High | 6.5 (5.3 – 8.0) *** | 7.7 (5.5 – 11.0) *** | 6.2 (5.1 – 7.6) *** | 7.2 (4.9 – 10.4) *** | 6.3 (5.2 – 7.7) *** | 6.7 (5.0 – 8.9) *** |
| 2 | Education (ref: Primary) |  |  |  |  |  |  |
|  | Secondary | 0.8 (0.7 – 0.9) *** | 0.9 (0.7 – 1.0) * |  |  |  |  |
|  | College | 0.6 (0.5 – 0.8) *** | 0.8 (0.6 – 1.2) |  |  |  |  |
|  | Life event#Education |  |  |  |  |  |  |
|  | Moderate#Secondary |  | 0.8 (0.6 – 0.9) * |  |  |  |  |
|  | Moderate#College |  | 0.5 (0.3 – 0.8) * |  |  |  |  |
|  | High#Secondary |  | 0.7 (0.4 – 1.1) |  |  |  |  |
|  | High#College |  | 0.9 (0.5 – 1.8) |  |  |  |  |
| 3 | Asset index  (ref: Lower asset) |  |  |  |  |  |  |
|  | Higher asset |  |  | 0.9 (0.8 – 1.1)* | 1.0 (0.8 – 1.2) |  |  |
|  | Life event#Asset index |  |  |  |  |  |  |
|  | Moderate#Higher asset |  |  |  | 0.8 (0.6 – 1.0)* |  |  |
|  | High#Higher asset |  |  |  | 0.8 (0.5 – 1.2) |  |  |
| 4 | Residency (ref: Rural) |  |  |  |  |  |  |
|  | Urban |  |  |  |  | 0.8 (0.7 – 0.9) *** | 0.9 (0.7 – 1.0) |
|  | Life event#Residency |  |  |  |  |  |  |
|  | Moderate#Urban |  |  |  |  |  | 0.7 (0.6 – 0.9) * |
|  | High#Urban |  |  |  |  |  | 0.6 (0.5 – 1.3) |
| 5 | Age (ref 18-24 years) |  |  |  |  |  |  |
|  | 25-34 | 0.8 (0.7 – 1.0) | 0.8 (0.7 – 1.0) * | 0.8 (0.7 – 1.0) | 0.8 (0.7 – 1.0) | 0.8 (0.7 – 1.0) | 0.8 (0.7 – 1.0) |
|  | 35-44 | 0.8 (0.7 – 1.0) | 0.8 (0.6 – 1.0) | 0.9 (0.7 – 1.1) | 0.9 (0.7 – 1.1) | 0.9 (0.7 – 1.1) | 0.9 (0.7 – 1.1) |
|  | 45-54 | 0.8 (0.6 – 0.9) | 0.8 (0.6 – 0.9) * | 0.8 (0.7 – 1.0) | 0.8 (0.7 – 1.0) | 0.8 (0.7 – 1.0) | 0.8 (0.7 – 1.0) |
|  | 55-64 | 0.7 (0.6 – 0.9) | 0.7 (0.6 – 0.9) * | 0.8 (0.6 – 1.0) | 0.8 (0.6 – 1.0) | 0.8 (0.6 – 1.0) | 0.8 (0.7 – 1.0) |
|  | 65-74 | 0.9 (0.7 – 1.2) | 0.9 (0.7 – 1.2) | 1.0 (0.8 – 1.4) | 1.0 (0.8 – 1.4) | 1.0 (0.8 – 1.4) | 1.0 (0.8 – 1.4) |
|  | ≥75 | 0.9 (0.6 – 1.3) | 0.9 (0.6 – 1.4) | 1.1 (0.7 – 1.6) | 1.1 (0.7 – 1.6) | 1.0 (0.7 – 1.5) | 1.1 (0.7 – 1.6) |
| 6 | Sex (ref male) |  |  |  |  |  |  |
|  | Female | 1.5 (1.3 – 1.6) *** | 1.5 (1.3 – 1.6) *** | 1.5 (1.3 – 1.6) *** | 1.5 (1.3 – 1.7) *** | 1.5 (1.3 – 1.7) *** | 1.5 (1.3 – 1.7) *** |
| 7 | Marital status (ref: Single) |  |  |  |  |  |  |
|  | Married | 0.6 (0.5 – 0.8) *** | 0.7 (0.5 – 0.8) *** | 0.6 (0.5 – 0.8) *** | 0.7 (0.5 – 0.8) *** | 0.6 (0.5 – 0.8) *** | 0.6 (0.5 – 0.8) *** |
|  | Widowed | 0.7 (0.5 – 0.9) * | 0.7 (0.5 – 0.9) * | 0.7 (0.5 – 0.9) * | 0.7 (0.5 – 0.9) * | 0.7 (0.5 – 0.9) * | 0.7 (0.5 – 0.9) * |
|  | Divorced | 0.9 (0.6 – 1.3) | 0.9 (0.6 – 1.3) | 0.9 (0.6 – 1.3) | 0.9 (0.6 – 1.3) | 0.9 (0.6 – 1.3) | 0.9 (0.6 – 1.3) |
| 8 | Province (ref: West Java) |  |  |  |  |  |  |
|  | Central Java | 1.2 (1.0 – 1.4) ** | 1.2 (1.0 – 1.4) * | 1.1 (1.0 – 1.3) | 1.1 (1.0 – 1.3) | 1.2 (1.0 – 1.4) * | 1.2 (1.0 – 1.4) * |
|  | East Java | 1.0 (0.9 – 1.2) | 1.0 (0.9 – 1.2) | 1.0 (0.8 – 1.1) | 1.0 (0.8 – 1.1) | 1.0 (0.8 – 1.0) | 0.9 (0.8 – 1.1) |
|  | Banten | 1.0 (0.9 – 1.3) | 1.0 (0.8 – 1.2) | 0.9 (0.7 – 1.1) | 0.9 (0.7 – 1.1) | 1.0 (0.8 – 1.3) | 1.0 (0.8 – 1.3) |
|  | Log Likelihood | -5268.6 | -3917.9 | -5276.3 | -5273.6 | -5269.1 | -5265.4 |
|  | Nagelkerke Pseudo-R2 | 0.069 | 0.070 | 0.067 | 0.068 | 0.069 | 0.070 |
|  | AIC | 0.54 | 0.55 | 0.55 | 0.55 | 0.55 | 0.55 |
|  | Hosmer-Lemeshow Chi2 | 3.1 | 10.0 | 4.6 | 3.9 | 23.0 | 20.6 |
|  | Observations | 19,123 | 19,123 | 19,132 | 19,132 | 19,132 | 19,132 |

Note: AOR=Adjusted Odds Ratio; CI =Confidence Interval in parenthesis; ***p < 0.001, **p < 0.005, * p < 0.05. Model a: without interaction, Model b: with interaction
