# Supplementary material for: The association between life events and mental health among adults in Java, Indonesia: Investigating the moderating effects by education, asset index, and rural-urban area of residence
Source: PLoS One. 2026 May 18;21(5):e0348726. doi: 10.1371/journal.pone.0348726 (PMC13183217; doi:10.1371/journal.pone.0348726)
Supplement: S4 Table — (DOCX) [file pone.0348726.s005.docx]

**S4 Table. Descriptive information of each province**

|  | **Information** | **Banten** | **West Java** | **Central Java** | **East Java** |
| --- | --- | --- | --- | --- | --- |
| 1 | Area (km^2^) | 9,352.77 | 37,044.86 | 34,377.49 | 48,036.84 |
| 2 | Population in 2025 (in thousand) | 12,537.4 | 50,759 | 38,233.9 | 42,089.3 |
| 3 | Percentage of poor people in 2024 (%) | 5.70 | 7.08 | 9.58 | 9.56 |
| 4 | Number of poor people in 2024 (in thousands) | 777,49 | 3,668.35 | 3,396.34 | 3,893.82 |
| 5 | Labour Force Participation Rate in 2024 | 66.17 | 67.71 | 73.74 | 73.45 |
| 6 | Provincial Minimum Wages per Month in 2024 (in IDR) | 2,727,812 | 2,057,495 | 2,036,947 | 2,165,244 |
| 7 | Percentage of dwelling ownership (%) | 86.49 | 83.44 | 90.90 | 90.93 |
| 8 | Percentage of private-toilet ownership (%) | 92.76 | 89.82 | 91.04 | 88.12 |
| 9 | Community Literacy Development Index in 2024 | 61.88 | 72.76 | 70.57 | 78.60 |
| 10 | Happiness index | 68.08 | 70.23 | 71.73 | 72.08 |

Source: BPS-Statistics Indonesia 2025 [1]

**Reference:**

1. BPS-Statistics Indonesia. Statistical Yearbook of Indonesia Jakarta: Directorate of Statistical Dissemination; 2025. Available from: <https://www.bps.go.id/en/publication/2025/02/28/8cfe1a589ad3693396d3db9f/statistical-yearbook-of-indonesia-2025.html>.
